# *Ulva prolifera* Polysaccharide–Manganese Alleviates Inflammation and Regulates Microbiota Composition in Dextran Sulfate Sodium-Induced Colitis Mice

**DOI:** 10.3389/fmicb.2022.916552

**Published:** 2022-05-24

**Authors:** Haoran Xue, Wei Song, Zongling Wang, Qian Wang

**Affiliations:** ^1^Department of Clinical Laboratory, Qilu Hospital (Qingdao) of Shandong University, Qingdao, China; ^2^Ministry of Natural Resources (MNR) Key Laboratory of Marine Eco-Environmental Science and Technology, First Institute of Oceanography, Ministry of Natural Resources, Qingdao, China; ^3^Department of Clinical Laboratory, Qilu Hospital (Jinan) of Shandong University, Jinan, China

**Keywords:** colitis, inflammation, manganese, microbe, polysaccharide

## Abstract

Manganese (Mn) deficiency exacerbates colitis symptoms, whereas diet supplemented with inorganic Mn merely maintains colon length in experimental colitis. In the present study, a new form of Mn, *Ulva* prolifera polysaccharide cheated-Mn (PMn) was used and its treatment effects on dextran sulfate sodium (DSS)-induced colitis were investigated. Male C57BL/6 mice were orally administrated with 3.5% DSS to induce colitis. Then, the colitis mice were treated with PBS or PMn for 7 days. The results showed that PMn administration help retrieve the body weight loss and intestinal morphology damage, and alleviate apoptosis and inflammatory responses in colitis mice. Moreover, PMn administration decreased intestinal infiltration as indicated by decreased concentration of myeloperoxidase and eosinophil peroxidase. Importantly, PMn retrieved the increased abundance of Firmicutes and the decreased abundance of Bacteroidetes caused by DSS, suggested its beneficial roles in regulating microbiota composition in mice with colon inflammation. Gut microbiota composition at the genus level in the mice administrated with PMn was similar to those in control mice, whereas they were clearly distinct from DSS-treated mice. These results support the potential therapeutic role of PMn in the treatment of intestinal colitis and microbes may play critical roles in mediating its effects.

## Introduction

Crohn's disease and ulcerative colitis are important types of inflammatory bowel diseases (IBD), which are characterized by chronic inflammatory condition (Zhang et al., 2018). For decades, IBD is becoming popular worldwide and the incidence is promptly growing. Except the over-accumulation of inflammatory cytokines, adverse changes such as impairment of intestinal morphology and barrier function, increased infiltration and dysbiosis of the gut microbiota are associated with the development of the disease (Munyaka et al., 2016; He et al., 2022). However, although the mechanisms related to IBD are studied and various therapies aimed to decrease inflammatory responses and retrieve gut microbial homeostasis are explored, ideal cure for IBD remains limited.

Trace minerals such as manganese (Mn), zinc (Zn) and selenium (Se) have been proved to exert beneficial effects on dextran sulfate sodium (DSS)-induced colitis (Tran et al., 2007; Nettleford and Prabhu, 2018; Choi et al., 2020). Meanwhile, a deficiency of one of these trace minerals could exacerbate experiment colitis (Iwaya et al., 2011; Choi et al., 2020). Specifically, Mn as an essential micronutrient required for bone growth, maintenance of cellular energy and oxidative status (Horning et al., 2015), has been proved to enhance tolerance to experiment colitis to a certain degree (Choi et al., 2020). Notably, Mn deficiency exacerbates colitis as indicated by increased weight loss, worse histopathological damage and over-accumulated inflammatory cytokines in the intestine (Choi et al., 2020). These results suggest that Mn is critical for the maintenance of intestinal function.

*Ulva prolifera* is a green alga that is widely flourishing in the Bohai sea of China. The extracts of *Ulva prolifera* is rich in polysaccharides, which has many beneficial effects including alleviating oxidative stress and inflammatory response, and improving insulin resistance (Song et al., 2018; Feng et al., 2020). Polysaccharides with negative charges can bind to cations such as Fe^2+^, Cu^2+^, Zn^2+^ and Mn^2+^, making them to be potentially used as chelating substances (Ferreira et al., 2015; Pang et al., 2015). Notably, *Ulva prolifera* polysaccharides has strong chelating ability and its chelating complex with trace mineral are stable and effective (Chi et al., 2018). Although the chelates of polysaccharide with Zn showed anti-inflammatory effect (Zhao et al., 2017), the application of *Ulva* polysaccharide-Mn in the treatment of IBD remains unexplored. Consequently, the present study was conducted to explore the effects of *Ulva* polysaccharide-Mn on body weight, intestinal morphology and inflammation in a mice model of DSS-induced colitis. Furthermore, whether microbes were involved in and mediated the beneficial effects of *Ulva* polysaccharide-Mn were also explored.

## Materials and Methods

### Animal Experiments

The experimental protocol was approved by the Protocol Management and Review Committee of the First Institute of Oceanography of China, and the mice were cared for and sacrificed according to the animal care guidelines of the First Institute of Oceanography of China.

Male C57BL/6j mice at the age of 6 weeks were acclimated for 1 week and then randomly divided into four groups. All animals were maintained in plastic cages at 22 ± 2°C with a relative humidity of 50 ± 5%. Meanwhile, they had free access to feed and water. Those mice drunk fresh running water for 7 days and then orally administrated with 0.1 mL PBS for another 7 days were designated as the control group (CON, *n* = 7); mice drunk water containing 3.5% DSS (wt/vol, molecular weight, 36,000–50,000 Da; MP Biomedicals, Shanghai, China) for 7 days and then orally administrated with 0.1 mL PBS for another 7 days were designated as the DSS-treated group (DSS, *n* = 7); mice drunk water containing 3.5% DSS for 7 days and then orally administrated with *Ulva* polysaccharide-Mn (Mn, 100 ppm) dissolved in 0.1 mL PBS for another 7 days were designated as the low Mn-treated group (LPMn, *n* = 7); mice drunk water containing 3.5% DSS for 7 days and then orally administrated with *Ulva* polysaccharide-Mn (Mn, 300 ppm) dissolved in 0.1 mL PBS for another 7 days were designated as the high Mn-treated group (HPMn, *n* = 7). *Ulva prolifera* was collected from the coast of Beidaihe in the Bohai Sea in May 2021. Polysaccharide was extracted and *Ulva* polysaccharide-Mn complex was prepared as described previously (Cho et al., 2011; Li et al., 2016). At the end of the experiment on day 14, all the animals were euthanized by cervical dislocation. Then, the colonic tissue and the content in the colon were immediately collected and stored at −80°C for further analysis.

### Assessment of Disease Activity

Colitis development was observed daily by recording the body weight change and the disease activity index (DAI) using a standard scoring system as described previously (Zhang et al., 2018). Briefly, no weight loss scored as 0; 0.1–5% scored as 1; 5–10% scored as 2; >10% scored as 3. Stool consistency: well-formed pellets scored as 0; semi-formed stools scored as 1; liquid stools scored as 2. Rectal bleeding: no blood scored as 0; small amount of blood as 1; blood regularly observed scored as 2; blood in all stool scored as 3.

### Real-Time Quantitative PCR (RT-qPCR)

Total RNA was extracted from colonic tissue by using the TRIzol reagents (Invitrogen, Shanghai, China). RNA samples were then used to generate cDNA by using the Reverse Transcription reagent Kit (Thermo Scientific, Shanghai, China). Then, RT-qPCR was performed as described previously (Zhou et al., 2021). Relative mRNA expression of the target genes was calculated as the ratio to the mRNA level of the β-actin gene using the 2^−Δ*ΔCt*^ method. All primers used were presented in Supplementary Table 1.

### Determination of Myeloperoxidase (MPO), and Eosinophil Peroxidase (EPO) Levels in the Colon

MPO and EPO levels in the colonic tissue were analyzed using commercial ELISA quantitative kits (Boyan, Nanjing, China) according to the manufacturer's instructions. Protein concentration was quantified by using BCA assay kit (Beyotime, Shanghai, China).

### Histological Analyses

The colons (2.0–3.0 cm proximal to the anus) were collected for the observation of histological changes as previously described (Zhou et al., 2018). Briefly, they were excised and softly washed with ice-cold PBS. Then, the samples were fixed in 4% paraformaldehyde and embedded in paraffin after they were opened longitudinally. Finally, the samples were sliced into section and performed with Hematoxylin-Eosin (HE) staining.

### Assessment of Apoptosis

The samples of colonic tissue (1.0–2.0 cm proximal to the anus) were collected for the determination of apoptosis as previously described (He et al., 2020). Briefly, the embedded samples were sliced into 5-μm thickness sections and stained using an *in situ* cell death detection kit (Roche, Darmstadt, Germany) and then representative pictures were obtained.

### Gut Microbiota Profiling

Colonic content was used for DNA extraction and isolation with the QIAampDNA stool Mini Kit (QIAGEN, Beijing, China). The V3–V4 region of the bacterial 16S rDNA gene sequences were amplified using primers of 5'-CCTAYGGGRBGCASCAG-3' (341Forward) and 5'-GGACTACNNGGGTATCTAAT-3' (806Reverse). Then, PCR analysis were performed as previously described (Zhang et al., 2018) and amplicons were sequenced using the Illumina HiSeq2500 platform. Representative operational taxonomic units were used for analysis using the Greengenes database.

### Statistics Analysis

Significance between groups was analyzed using one-way ANOVA followed by Student-Newman-Keuls *post hoc* test, using the SPSS Statistics 18.0 Software. All results are expressed as mean ± SEM. *P* value < 0.05 was considered significant.

## Results

### *Ulva* Polysaccharide-Mn Alleviated DSS-Induced Colitis Symptoms

**Figure 1:**
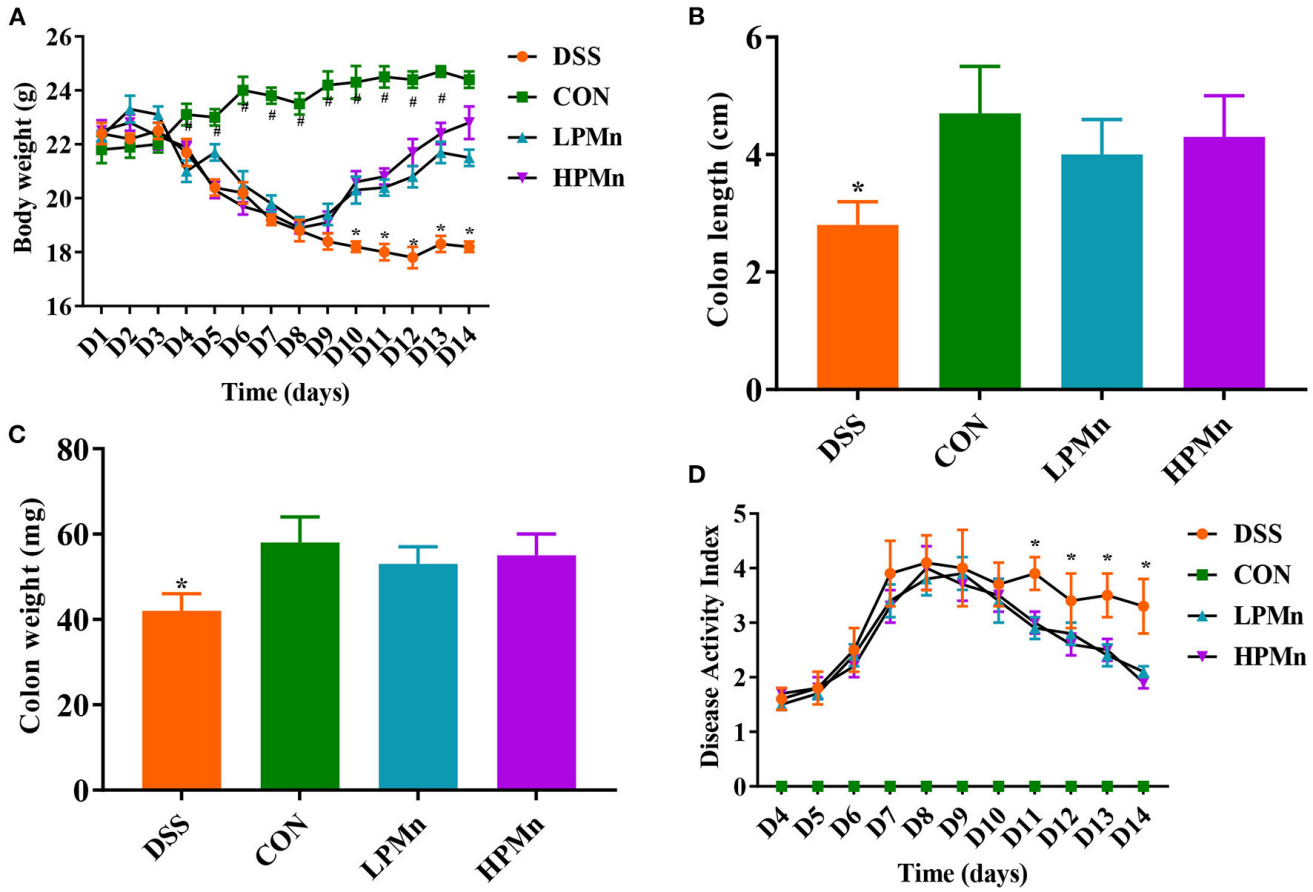
*Ulva* polysaccharide-manganese alleviated DSS-induced colitis symptoms. **(A)** Body weight change. **(B)** Colon length. **(C)** Colon weight. **(D)** Disease activity index. Values are expressed as mean ± SEM, *n* = 7. *P < 0.05 (Significant difference between DSS group and other three groups); ^#^*P* < 0.05 (Significant difference between CON group and other three groups).

As shown in Figure 1, DSS treatment induced significant body weight loss from day 4. *Ulva* Polysaccharide-Mn supplementation led to the recovery of body weight on day 10 (Figure 1A). At the end of the experiment, no significant difference in body weight were observed between mice in the CON and HPMn group. Colon length and weight were lower in DSS-treated mice than in control mice and in *Ulva* polysaccharide-Mn treated mice (Figures 1B,C). During the experiment, *Ulva* polysaccharide-Mn supplementation decreased DAI from day 11 to day 14 (Figure 1D).

### *Ulva* Polysaccharide-Mn Improved Inflammatory Response and Colonic Infiltration

**Figure 2:**
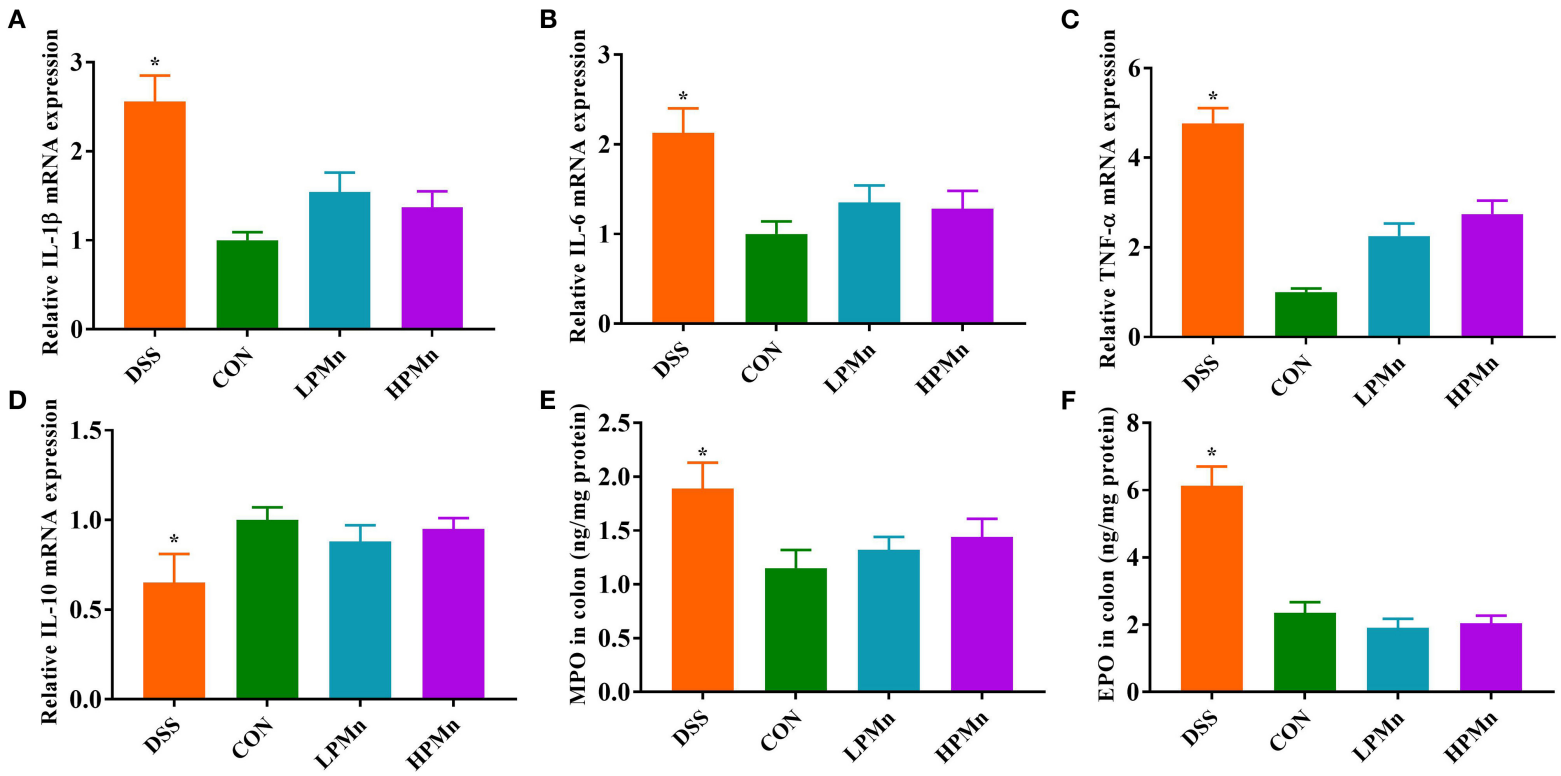
*Ulva* polysaccharide-manganese improved inflammatory response and colonic infiltration. Relative mRNA expression of *IL-1*β **(A)**, *IL-6* **(B)**, *TNF-*α **(C)** and *IL-10* **(D)**. Colonic concentration of MPO **(E)** and EPO **(F)**. Values are expressed as mean ± SEM, *n* = 7. *P < 0.05 (Significant difference between DSS group and other three groups).

As shown in Figure 2, gene expression of *IL-1*β, *IL-6* and *TNF-*α in the colon of DSS-treated mice were significantly higher, whereas *IL-10* gene expression was significantly lower, when compared with those in the other three treatment groups. DSS intervention induced increases in colonic level of MPO and EPO, while *Ulva* polysaccharide-Mn supplementation alleviated such changes.

### *Ulva* Polysaccharide-Mn Improved Histopathological Damage and Apoptosis

**Figure 3:**
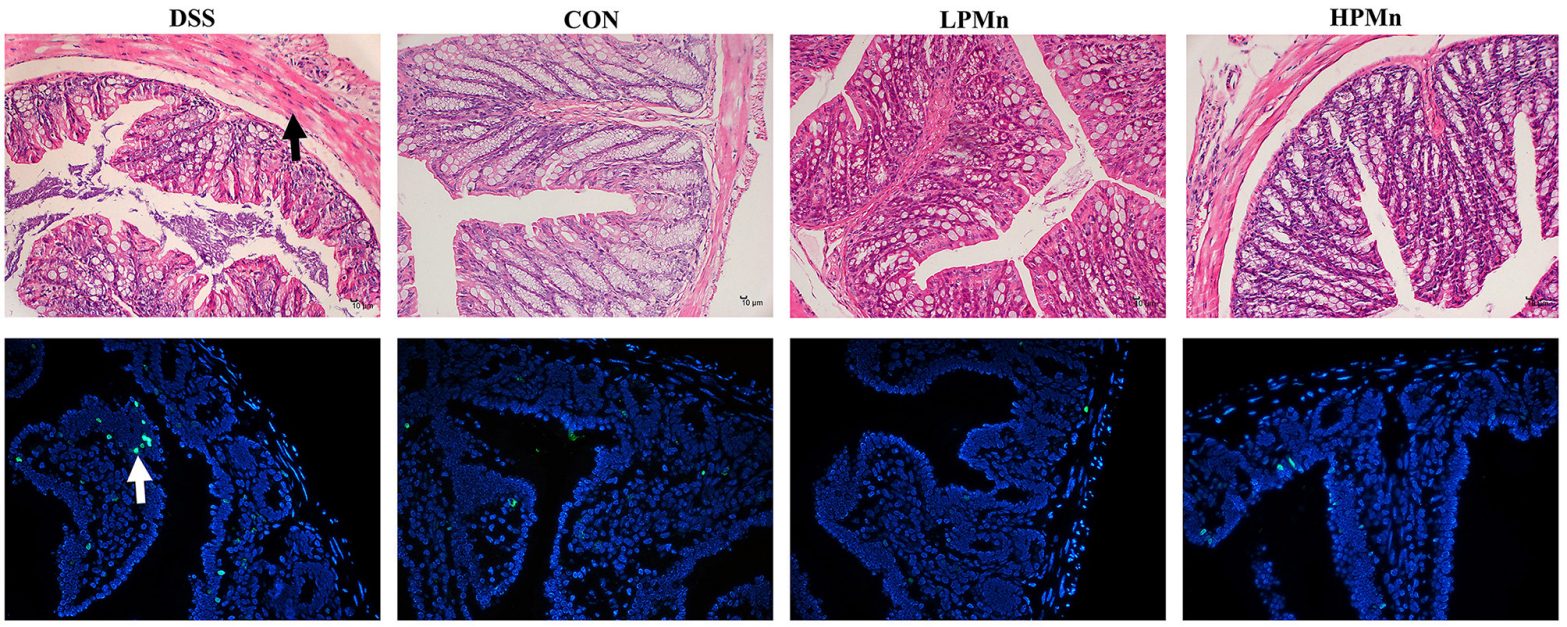
*Ulva* polysaccharide-manganese improved histopathological damage and apoptosis. Upper, representative images of HE staining results (Arrow, morphology damage); Lower, representative images of TUNEL staining results (Arrow, apoptosis cells).

As shown in Figure 3, the colon of DSS-treated mice showed edema and shedding, while control mice and mice with *Ulva* polysaccharide-Mn supplementation had no such damage. TUNEL staining showed that DSS induced high level of apoptosis in the colonic tissue whereas *Ulva* polysaccharide-Mn administration alleviated this change.

### *Ulva* Polysaccharide-Mn Altered Microbial Diversity

**Figure 4:**
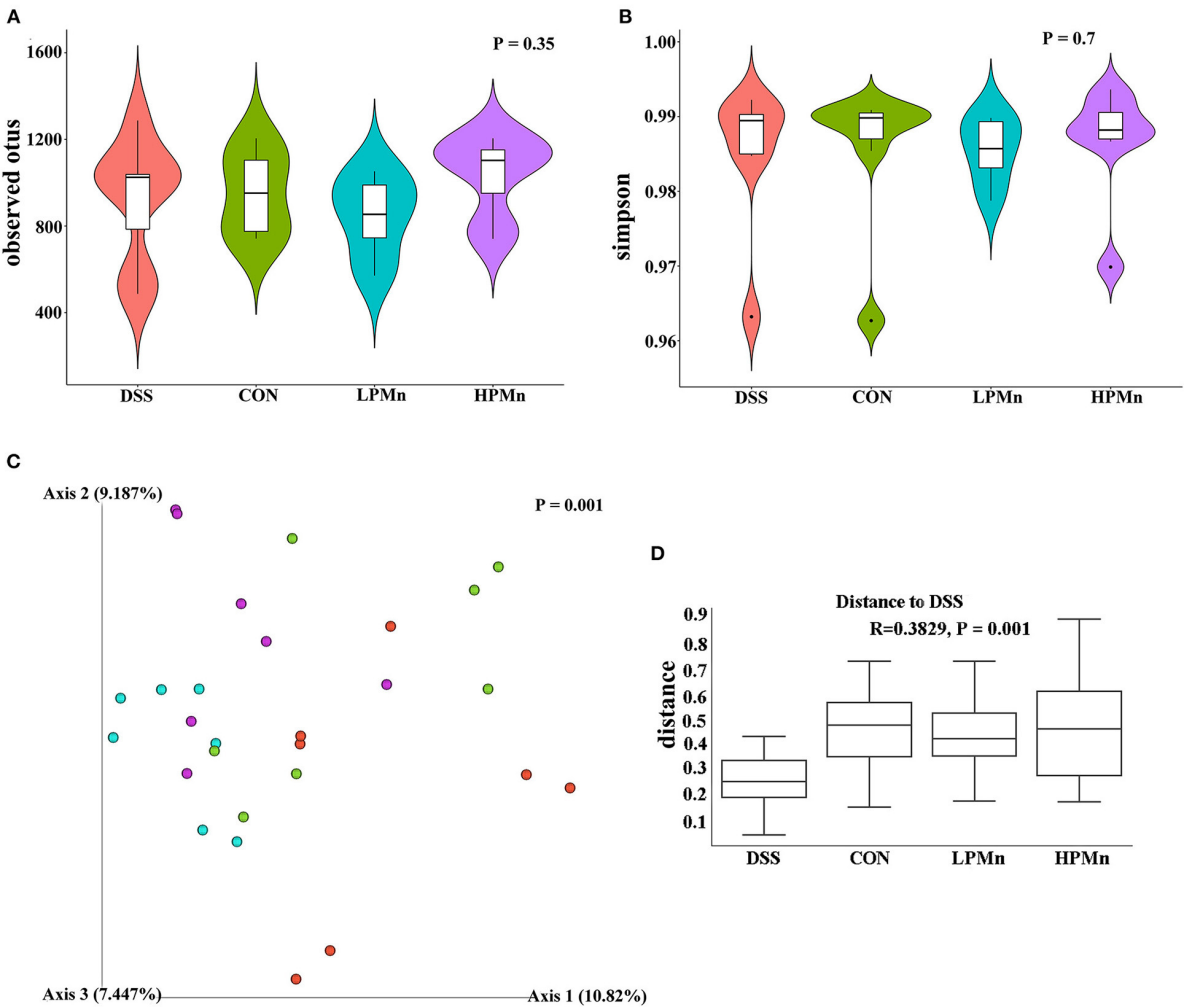
*Ulva* polysaccharide-manganese altered microbial diversity. **(A)** Observed otus. **(B)** Simpson. **(C)** PCoA plot of the microbiota based on an unweighted UniFrac metric. **(D)** Results of anosim analysis.

As shown in Figure 4, the alpha diversity as indicated by the index of observed otus and simpson, was not significantly changed among all the treatments (Figures 4A,B). Beta-diversity showed significant difference among the four treatment groups as indicated by the unweighted Unifrac PCoA results, which showed that the microbial structure in the colonic content of mice from both the HPMn and LPMn groups were separated from the DSS-treated mice (Figure 4C). Additionally, anosim analysis also showed significant difference among all the groups (Figure 4D).

### *Ulva* Polysaccharide-Mn Altered Microbial Composition and Microbiome Phenotypes

**Figure 5:**
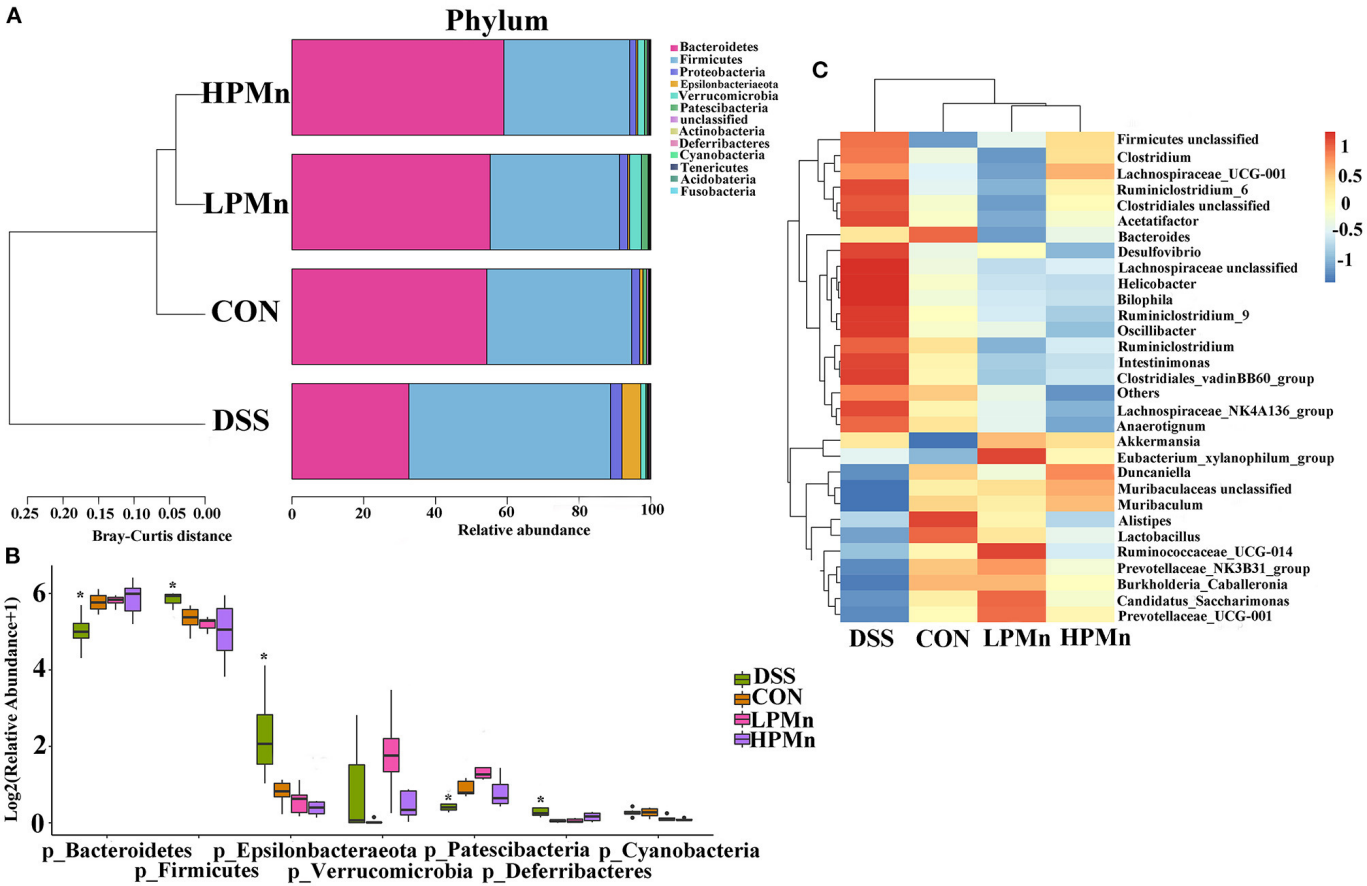
*Ulva* polysaccharide-manganese altered microbial composition. **(A,B)** Relative abundance of predominant bacteria at the phylum level. **(C)** Relative abundance of predominant bacteria at the genus level. Values are expressed as mean ± SEM, *n* = 7. *P < 0.05 (Significant difference between DSS group and other three groups).

As showed in Figure 5, the results showed that Bacteroidetes and Firmicutes were the main phyla in the colonic microbial population. Bacteroidetes abundance was significantly decreased whereas Firmicutes abundance was significantly increased by DSS treatment. No significant change in Bacteroidetes and Firmicutes abundance were observed among the mice in the CON, LPMn and HPMn groups. Additionally, Epsilonbacteraeota and Deferribacteres abundance were significantly increased whereas Patescibacteria abundance was significantly decreased in mice in the DSS group, when compared with those in the CON, LPMn and HPMn groups. DSS-induced alteration in microbial composition was characterized by high abundance of *Clostridium, Ruminiclostridium, Lachnospiraceae, Helicobacter* and *Oscillibacter*, and low abundance of *Muribaculum, Lactobacillus* and *Prevotellaceae*. *Ulva* polysaccharide-Mn administration retrieved these changes induced by DSS.

**Figure 6:**
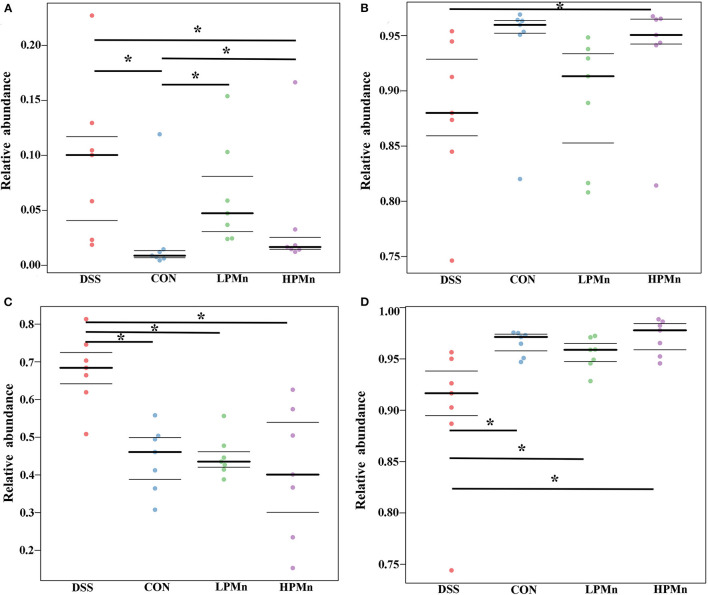
*Ulva* polysaccharide-manganese altered microbiome phenotypes. Relative abundance of aerobic **(A)** and anaerobic microbes **(B)**, microbes contains mobile elements **(C)**, and stress tolerant-related microbes **(D)**. Values are expressed as mean ± SEM, *n* = 7. *P < 0.05.

As shown in Figure 6, organism-level microbiome phenotypes were predicted using Bugbase. Aerobic microbes were significantly lower in the colonic content of mice in the CON group when compared with those in the other groups, whereas they were significantly higher in the mice of DSS group when compared with those in the HPMn group (Figure 6A). Anaerobic microbes were significantly lower in the colonic content of mice in the DSS group when compared with those in the HPMn group, whereas no significant change was observed among other groups (Figure 6B). Microbes in DSS-treated mice showed increased mobile elements (Figure 6C) and decreased stress tolerance (Figure 6D) when compared with those in the other three groups, whereas no significant difference was observed among other groups.

## Discussion

Although dietary supplementation with Mn was reported to ameliorate DSS-induced colitis, it merely help maintain the colon length. Notably, dietary Mn had no effect on the composition of microbes, which play critical roles during the development of inflammation (Choi et al., 2020). In the present study, we used the *Ulva* polysaccharide-Mn and found that it could retrieve colitis as indicated by the recovery of body weight and intestinal morphology, decreased expression of inflammatory cytokines, improved gut microbiota composition. Our results suggested that *Ulva* polysaccharide and Mn might exert synergetic effects on intestinal inflammation and dysbiosis of gut microbiota.

Colitis is firstly characterized with increased gene expression of inflammatory cytokines and over-production of pro-inflammatory cytokines, which indicated a chronic inflammatory status. These changes cause damage of intestinal permeability and result in increased infiltration in the colon (Hering and Schulzke, 2009). Extracts from *Ulva prolifera* could alleviate inflammation response in difference conditions (Song et al., 2018; Feng et al., 2020), suggested its potential role in restoring intestinal impairment caused by inflammation (Chen et al., 2006). Meanwhile, Mn could also regulate gene expression of pro-inflammatory cytokines. The remarkable effects of *Ulva* polysaccharide-Mn on gene expression of *IL-1*β, *IL-6, TNF-*α and *IL-10* in our results confirmed the synergetic effects of polysaccharide and Mn. Importantly, DSS-induced polymorphonuclear leukocytes and eosinophils infiltration as indicated by increased colonic MPO and EPO concentration, which was in line with previous studies (Zhang et al., 2015, 2018). Yet, administration with *Ulva* polysaccharide-Mn help recover their concentrations, which could be result from the relaxation of inflammatory status. According to our results, *Ulva* polysaccharide-Mn showed better effects on colonic inflammation in comparison to inorganic Mn as previously described (Choi et al., 2020). However, no common target involved in inflammation responses for polysaccharide and Mn were reported. Future studies are required to explore the mechanisms related to the synergetic effects of polysaccharide and Mn on intestinal inflammation.

Microbiota play critical roles in the development of colitis. Importantly, colitis induced by DSS showed dysbiosis of gut microbiota. *Ulva* polysaccharide-Mn retrieved the increased abundance of Firmicutes and the decreased abundance of Bacteroidetes, suggested its beneficial roles in regulating microbiota composition in mice with colon inflammation. Notably, gut microbiota composition at the genus level in the mice administrated with *Ulva* polysaccharide-Mn was similar to those in control mice, whereas they were clearly distinct from DSS-treated mice. *Ulva* polysaccharide-Mn decreased the abundance of Clostridia which is usually considered as virulent organisms and spore formers (Finegold, 2011). Meanwhile, it also decreased the abundance of *Desulfovibrio*, an anaerobic bacillus produces important virulence factors (Finegold, 2011). These results suggested that *Ulva* polysaccharide-Mn reduced the potential threat of harmful microbes. *Ulva* polysaccharide-Mn increased the abundance of *Lactobacillus*, most of which are widely considered as beneficial microbes. Moreover, *Ulva* polysaccharide-Mn restored the abundance of *Prevotellaceae*, which is exhausted in inflammatory conditions and is negatively associated with colitis (Shi et al., 2021). Surprisingly, the abundance of *Lachonospiraceae*, which is a commonly beneficial inhabitant, was increased by DSS treatment, whereas it was recovered by *Ulva* polysaccharide-Mn. The altered abundance of *Lachonospiraceae* could be affected by other microbes but not the direct effect of DSS and *Ulva* polysaccharide-Mn. All these results indicated that *Ulva* polysaccharide-Mn help recover the microbial homeostasis. We speculated that microbes may critically mediate the beneficial effects of *Ulva* polysaccharide-Mn on colitis, although the specific mechanisms need to be elucidated in the future works.

In conclusion, we demonstrated that *Ulva* polysaccharide-Mn had anti-inflammatory effects and retrieved the experiment colitis. Importantly, *Ulva* polysaccharide-Mn help recover the altered microbiota composition in colitis mice. These results support the potential therapeutic role of *Ulva* polysaccharide-Mn in the treatment of intestinal colitis.

## Data Availability Statement

The data presented in the study are deposited in the NCBI repository, accession number PRJNA832883 (www.ncbi.nlm.nih.gov/bioproject/PRJNA832883).

## Ethics Statement

The animal study was reviewed and approved by Protocol Management and Review Committee of the First Institute of Oceanography of China.

## Author Contributions

HX and QW designed the experiments. HX, WS, and ZW performed the experiments, analyzed the data, and drafted the manuscript. QW revised the manuscript. All authors contributed to the article and approved the submitted version.

## Funding

This work was financially supported by the Key R&D Program of Shandong Province under (2021CXGCHT01), Wu Jie Ping Medical Foundation (320.6750.18315), and Science and Technology Development Program of Qingdao City (19-6-1-20-nsh).

## Conflict of Interest

The authors declare that the research was conducted in the absence of any commercial or financial relationships that could be construed as a potential conflict of interest.

## Publisher's Note

All claims expressed in this article are solely those of the authors and do not necessarily represent those of their affiliated organizations, or those of the publisher, the editors and the reviewers. Any product that may be evaluated in this article, or claim that may be made by its manufacturer, is not guaranteed or endorsed by the publisher.
