# Supplementary material for: Ulva prolifera Polysaccharide–Manganese Alleviates Inflammation and Regulates Microbiota Composition in Dextran Sulfate Sodium-Induced Colitis Mice
Source: Front Microbiol. 2022 May 24;13:916552. doi: 10.3389/fmicb.2022.916552 (PMC9205199; doi:10.3389/fmicb.2022.916552)
Supplement: Supplementary file 1 [file Table_1.DOCX]

**Supplementary Table 1** Primer sequences

| Gene | 5’-3’ Primer sequence |
| --- | --- |
| TNF-α | F: ATGAGAAGTTCCCAAATGGC |
|  | R: CTCCACTTGGTGGTTTGCTA |
| IL-1β | F: TGCCACCTTTTGACAGTGATG |
|  | R: AAGGTCCACGGGAAAGACAC |
| IL-6 | F: CCTCTCTGCAAGAGACTTCCAT |
|  | R: AGTCTCCTCTCCGGACTTGT |
| IL-10 | F:GGACCAGCTGGACAACATACTGCTA |
|  | R: CCGATAAGGCTTGGCAACCCAAGT |
| β-actin | F: TGTCCACCTTCCAGCAGATGT |
|  | R: AGCTCAGTAACAGTCCGCCTAGA |
